# An open-label, non-randomized study of the pharmacokinetics of the nutritional supplement nicotinamide riboside (NR) and its effects on blood NAD+ levels in healthy volunteers

**DOI:** 10.1371/journal.pone.0186459

**Published:** 2017-12-06

**Authors:** Sophia E. Airhart, Laura M. Shireman, Linda J. Risler, Gail D. Anderson, G. A. Nagana Gowda, Daniel Raftery, Rong Tian, Danny D. Shen, Kevin D. O’Brien

**Affiliations:** 1 Division of Cardiology, Department of Medicine, University of Washington School of Medicine, Seattle, Washington, United States of America; 2 Department of Pharmaceutics, University of Washington School of Pharmacy, Seattle, Washington, United States of America; 3 Department of Pharmacy, University of Washington School of Pharmacy, Seattle, Washington, United States of America; 4 Northwest Metabolomics Research Center, Department of Anesthesiology & Pain Medicine; University of Washington, Seattle, Washington, United States of America; 5 Mitochondria and Metabolism Center, Department of Anesthesiology & Pain Medicine; University of Washington, Seattle, Washington, United States of America; Havard Medical School, UNITED STATES

## Abstract

**Objectives:**

The co-primary objectives of this study were to determine the human pharmacokinetics (PK) of oral NR and the effect of NR on whole blood nicotinamide adenine dinucleotide (NAD+) levels.

**Background:**

Though mitochondrial dysfunction plays a critical role in the development and progression of heart failure, no mitochondria-targeted therapies have been translated into clinical practice. Recent murine studies have reported associations between imbalances in the NADH/NAD+ ratio with mitochondrial dysfunction in multiple tissues, including myocardium. Moreover, an NAD+ precursor, nicotinamide mononucleotide, improved cardiac function, while another NAD+ precursor, nicotinamide riboside (NR), improved mitochondrial function in muscle, liver and brown adipose. Thus, PK studies of NR in humans is critical for future clinical trials.

**Methods:**

In this non-randomized, open-label PK study of 8 healthy volunteers, 250 mg NR was orally administered on Days 1 and 2, then uptitrated to peak dose of 1000 mg twice daily on Days 7 and 8. On the morning of Day 9, subjects completed a 24-hour PK study after receiving 1000 mg NR at t = 0. Whole-blood levels of NR, clinical blood chemistry, and NAD+ levels were analyzed.

**Results:**

Oral NR was well tolerated with no adverse events. Significant increases comparing baseline to mean concentrations at steady state (C_ave,ss_) were observed for both NR (*p* = 0.03) and NAD+ (*p* = 0.001); the latter increased by 100%. Absolute changes from baseline to Day 9 in NR and NAD+ levels correlated highly (R^2^ = 0.72, *p* = 0.008).

**Conclusions:**

Because NR increases circulating NAD+ in humans, NR may have potential as a therapy in patients with mitochondrial dysfunction due to genetic and/or acquired diseases.

## Introduction

Mitochondrial dysfunction has been implicated in multiple diseases, including heart failure [1–3]. However, there currently is no specific treatment for mitochondrial dysfunction in human heart failure or any other disease [4–7]. In recent years, there has been a surge of interest in targeting alterations in energy metabolism in heart failure. Development of mitochondria-based therapies for metabolic diseases has been hampered both by limited understanding of how mitochondrial impairment causes tissue dysfunction as well as by a lack of interventions shown to improve mitochondrial function. Recently, we demonstrated in a murine model that impaired mitochondrial oxidative phosphorylation led to an increased myocardial NADH/NAD+ ratio and increased mitochondrial protein acetylation without affecting mitochondrial production of reactive oxygen species (ROS) or synthesis of adenosine triphosphate (ATP) [8]. These changes rendered the heart susceptible to chronic stresses, which accelerated the development of heart failure. Similar increases in the NADH/NAD+ ratio and in protein acetylation also were seen in animal models of heart failure due to chronic pressure overload. Furthermore, intraperitoneal administration of the NAD+ precursor nicotinamide mononucleotide (NMN) to these mice normalized the NADH/NAD+ ratio, prevented the increase in mitochondrial protein acetylation, and improved cardiac function [9]. Promoting NAD+ synthesis through the salvage pathway by overexpressing nicotinamide phosphoribotransferase protected against ischemia-reperfusion injury and ischemic heart failure [10, 11]. These findings collectively suggest that augmentation of NAD+ could improve mitochondrial function and exert myocardial protection.

Nicotinamide riboside (NR) is a pyridine nucleoside form of vitamin B3 that is naturally found in milk and is available as a nutraceutical. NR is converted by nicotinamide riboside kinases (NRK1,2) to NMN, which is subsequently converted to NAD+ by nicotinamide mononucleotide adenylyltransferase (NMNAT). Oral supplementation with NR has been shown to increase NAD+ in brown adipose tissue, skeletal muscle and liver, and to improve mitochondrial function in a mouse model of diet-induced obesity [12]. Nutritional supplementation with NR, an NAD+ precursor, thus holds potential as an innovative therapy for heart failure and other disease states characterized by mitochondrial dysfunction. However, very limited information is available in regard to whether NR supplementation augments NAD+ levels in humans. Therefore, the primary objective of this study was to determine the pharmacokinetics of orally administered NR and its ability to increase blood NAD+ in healthy subjects following dose escalation of NR to 1000 mg twice daily. Primary outcomes were comparisons of baseline concentrations versus mean concentrations at steady state (C_ave,ss_) on Day 9 for NR and NAD+. Secondary outcomes were to determine the safety and tolerability of NR by assessing adverse event rates and comparisons of laboratory tests, specifically serum levels of potassium, creatine kinase (myositis), glucose (insulin resistance), uric acid and alanine aminotransferase.

## Methods

### Participants

**Fig 1:**
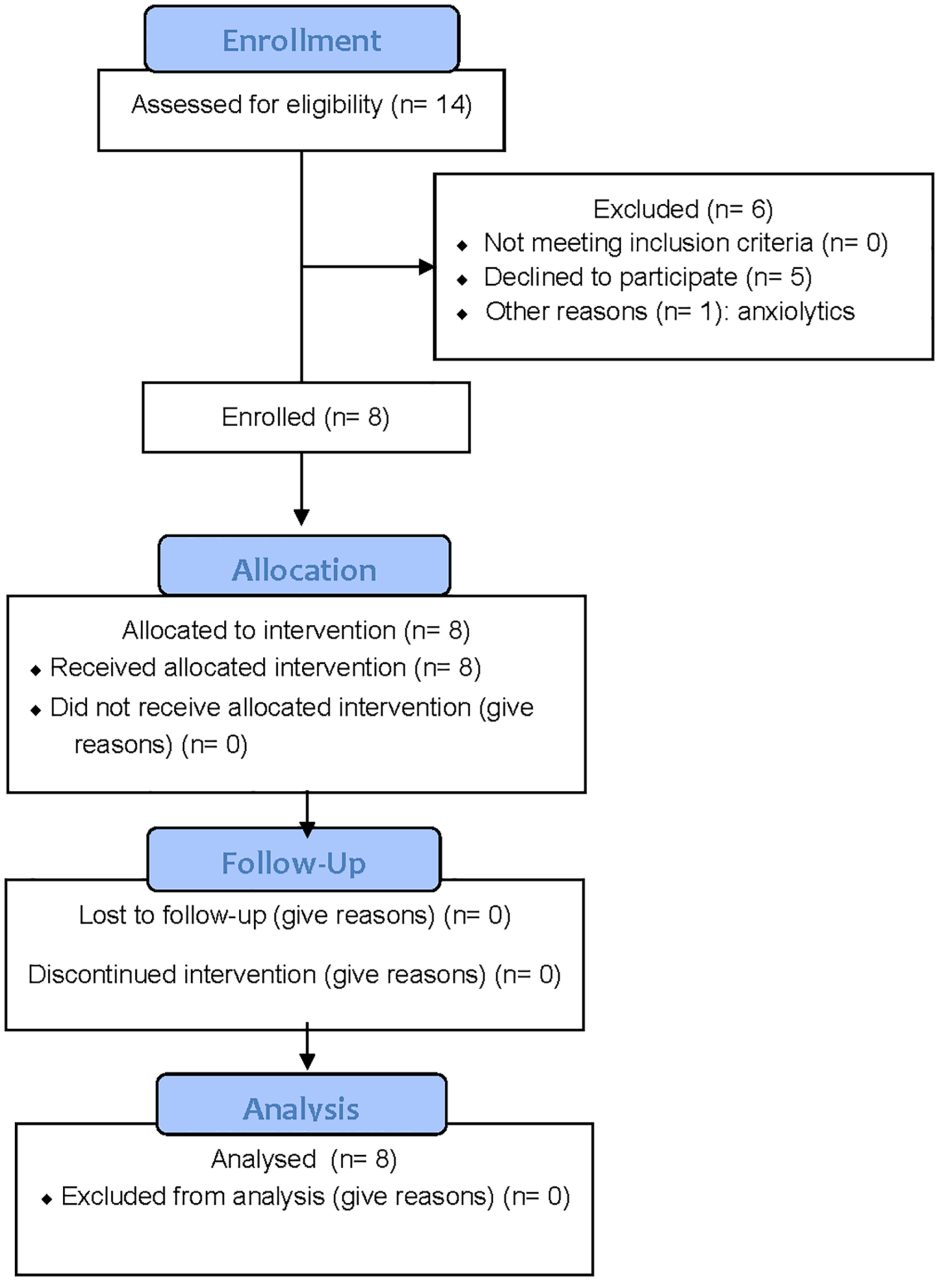
Study CONSORT flowchart.

The first participant was enrolled on November 4, 2015, and the final participant completed all study follow-up visits on December 23, 2015. Fourteen potential participants responded to a posting on the University of Washington (UW) Institute of Translational Health Sciences community volunteer forum (https://www.iths.org/community/for-volunteers-patients/). Five subsequently declined to participate and one was excluded due to anxiolytic medication use. Flow of participants through the Study is shown in Fig 1.

The remaining eight healthy volunteers (6 female, 2 male, age range 21–50 years) met all inclusion criteria and provided informed consent. None had any exclusion criteria, which included current smoking, pregnancy (i.e., positive urine pregnancy screening test in applicable females just prior to enrollment), current use of any medications or supplements (with the exception of birth control), any history of liver, renal, cardiovascular, endocrine or neurological disease, known allergy to niacin or nicotinamide riboside, or not being able to refrain from drinking alcohol during the duration of the study. All eight participants received the allocated intervention (NR), and all eight participants completed all Study procedures and follow-up. No participants were excluded from analyses. The standardized method for characterizing adverse events employed in the UW Clinical Trials Unit is the Common Terminology Criteria for Adverse Events v3.0 (CTCAE). There were no protocol deviations.

All research involving human participants was approved by the University of Washington Institutional Review Board (IRB) on August 4, 2015, and all clinical investigation was conducted according to the principles expressed in the Declaration of Helsinki. Written informed consent was obtained from all participants.

### Nicotinamide riboside: Source and administration schedule

**Fig 2:**
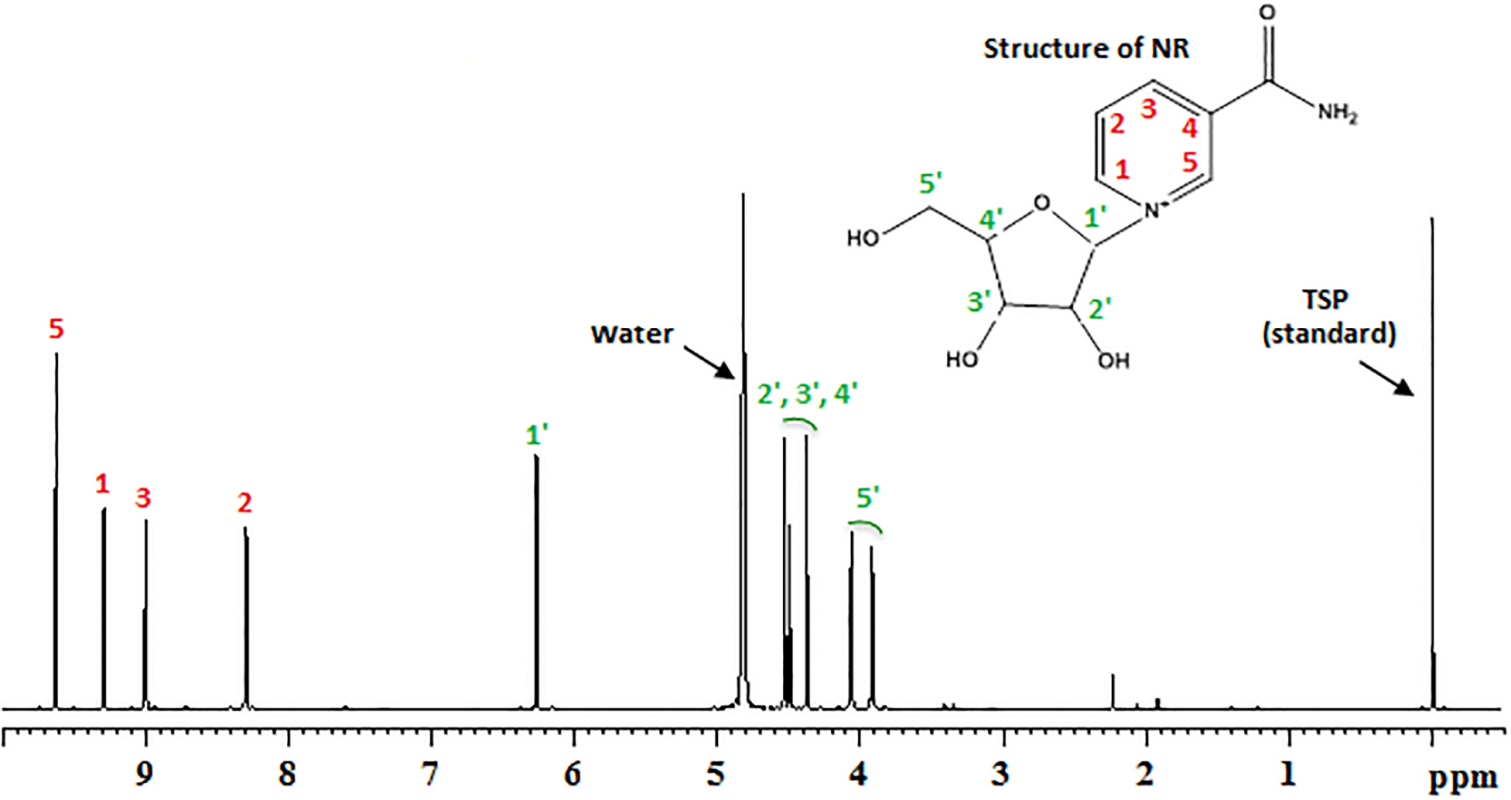
Typical ^1^H NMR spectrum of an aqueous extract (in deuterated water) of a NR capsule obtained on a bruker AVANCE III 800 MHz nuclear magnetic resonance (NMR) spectrometer. NMR signals that arise from NR are labeled with the corresponding location of the hydrogen atom(s) as shown in the molecular structure of NR (inset). TSP [3-(trimethylsilyl)propionic acid-2,2,3,3-d_4_ sodium salt] was used as an internal standard to quantify the amount and purity of NR in the capsules. The purity of NR was calculated based on integration of all the peaks and was in the range of 98–99%.

NR was supplied as 250 mg capsules by the manufacturer (Niagen^®^, ChromaDex, Irvine, CA). NR was manufactured in a GMP-compliant facility according to ISO/IEC 18025:2005 standards. Two Certificates of Analysis provided by the manufacturer and performed on separate lots reported ~99% purity of the NR preparation. In addition, an independent analysis was performed at the University of Washington on NR (Niagen^®^) purchased online (Amazon Prime, Amazon.com, Inc., Seattle, WA) by the investigators; 1H-NMR spectroscopy of this NR demonstrated 98–99% purity (Fig 2).

All participant clinic visits, medication dispensing and data collection were performed on the General Clinical Research Center (GCRC) at the University of Washington (UW). NR was self-administered by participants orally at 250 mg daily on Days 1 and 2, 250 mg twice daily on Days 3 and 4, 500 mg twice daily on days 5 and 6, and 1000 mg twice daily on Days 7 and 8. Once daily dosing was self-administered at approximately 8am. Twice daily dosing was self-administered at approximately 8am and 8pm.

On the morning of Day 9, participants were admitted to the UW GCRC for a 24-hour pharmacokinetic study. Participants were administered the final NR dose of 1000 mg at t = 0 by UW GCRC staff.

### Laboratory testing

Blood samples were obtained to monitor for potential side effects of NR therapy following 6-hour fasts and prior to NR dosing at baseline (Day 1) and on Days 2 and 9. Laboratory tests on these samples included complete blood count with white blood count differential and platelets, a serum chemistry panel (sodium, potassium, chloride, glucose, blood urea nitrogen, and creatinine), creatine kinase, aspartate aminotransferase (AST), alanine aminotransferase (ALT), uric acid, and lactate dehydrogenase.

NR and NAD+ concentrations were determined in blood samples drawn on Day 1 as well as in samples obtained on Day 9 at the following times after the last 1000 mg NR dose: 0.5, 1, 2, 3, 4, 6, 8, 12, 16, and 24 hours. Due to the reported presence of low levels of NR in milk, standardized meals devoid of milk and dairy products were provided during the 24-hour pharmacokinetic study.

NR and NAD+ assays were performed by the Pharmacokinetics Lab in the UW Department of Pharmaceutics. All other blood assays were performed by the Analytical Core of the UW Nutrition Obesity Research Center (NORC).

### Ethics

The study protocol followed the ethical standards of the University of Washington in accordance with the Helsinki Declaration of 1975 as revised in 1983. Recruitment for this study was conducted according to the Institutional Review Board (IRB) policies at the University of Washington, as well as Health Insurance Portability and Accountability Act (HIPAA) policies. All participants provided written, informed consent. The study has been registered on www.clinicaltrials.gov (Identifier: NCT02689882). A Data Safety Monitoring Board (DSMB) monitored ongoing safety of study participants. Safety monitoring included pre-specified evaluation for hyperkalemia, as well as for evidence for myositis (creatine kinase), dysglycemia (glucose), gout (uric acid), and hepatotoxicity (alanine aminotransferase). The DSMB reviewed participant-level and summary data at two time points: after study completion by the first 5 participants and again at end-of-study.

### Analysis of NR and NAD+ in whole blood

#### Materials

Acid citrate-dextrose (ACD) solution was purchased from Sigma-Aldrich (St. Louis, MO). The following reagents of analytical grade or higher were obtained from Sigma-Aldrich: NAD+, NMN, 8-bromoadenosine-5’-monophosphate (BMP), bovine serum albumin (BSA), citric acid, trichloroacetic acid, ammonium acetate, ammonium formate, methanol, and acetonitrile. The chloride salt of NR was purchased from ChromaDex, Inc. (Irvine, CA; Catalog #: ASB-00014313-001), and *d*_*4*_-NR triflate was purchased from Toronto Research Chemicals (Toronto, ON; Catalog #: N407772).

#### Blood sample collection

For NR analysis, 0.5 mL whole blood was transferred within 3 minutes after blood draw to a storage tube containing 0.070 mL ACD solution (22 g/L citric acid, trisodium salt; 7.3 g/L anhydrous citric acid; and 24.5 g/L *D*-(+)-glucose), 0.010 mL 2.5 M citric acid, and 200 ng of the internal standard, *d*_*3*_-NR. The mixture was vortexed briefly and immediately inserted into dry ice pending transfer to long-term storage at -80°C. For NAD+ analysis, whole blood was collected in Vacutainer^®^ tubes containing 0.105 M buffered sodium citrate (BD Diagnostics, Franklin Lakes, NJ), immediately placed on wet ice, and transferred to a -80°C freezer generally within 5 minutes after collection. Replicate aliquots were prepared and stored for each analysis to avoid the need for repeated freezing and thawing of the blood samples.

#### Assay procedure for NR

Calibration standards were prepared by adding known amounts of NR (2.5 ng to 20 ng) to tubes containing a solution of ACD, citric acid, and the internal standard *d*_*4*_-NR; 0.5 mL thawed frozen human blood was then added to each standard tube to achieve a final NR concentration range of 0.0195 μM to 0.156 μM. During preparation, all tubes were kept on wet ice. A 50 μL aliquot of each calibration standard was combined with 0.3 mL of 4% TCA, vortexed well, and put back on wet ice. Blood samples from the study were thawed quickly, one at a time, and then mixed well by vortexing. Fifty μL of each study sample were immediately combined with 0.3 mL of 4% TCA, vortexed and placed on wet ice. After protein precipitation was completed in all samples, the tubes were vortexed again and centrifuged for 10 minutes at 4°C and ~20,000 rcf. The supernatants were transferred to Costar Spin-X 0.22 μm spin filters (Corning; Corning, NY) and centrifuged for 1 minute at 4°C and ~20,000 rcf. The eluates were placed in a 96-well plate and 5 μL aliquots were injected onto an Agilent 1290 series ultra-performance liquid chromatograph coupled to an Agilent 6410 triple-quadrupole mass spectrometer (Agilent Technologies, Palo Alto, CA). Chromatographic separation of the analytes was achieved using a HyperCarb 100 x 2.1 mm, 5 μm particle-size column (Thermo Scientific). Mobile phase A was 10 mM ammonium formate, pH 3.5, and mobile phase B was methanol. The flow rate was 0.3 mL/min. The elution gradient started at 12.5% B, increased linearly to 15% B from 0.5 min to 1 min, then increased to 29% B by 2.5 min, and held at 29% B until 7.5 min. The mass spectrometer operated in electrospray ionization mode with positive polarity. The following ion transitions were monitored: 255.1 → 123.1 (NR) and 258.1 → 126.1 (*d*_*3*_-NR) *m/z*. The fragmentor voltage was set to 83 V, and the collision energy was 8 V. The capillary voltage was 1000 V.

#### Assay procedure for NAD+

An aliquot of frozen blood sample was thawed in a 30°C water bath for approximately 2 minutes; only 1–2 samples were thawed at a time. As soon as the blood had thawed, 50 μL were added to tubes containing 1 μg BMP as the internal standard and immediately followed by 300 μL of 4% trichloroacetic acid to precipitate proteins. Calibration standards were similarly prepared except that the matrix was 30 mg/mL BSA spiked with NAD+ and NMN that had been dissolved in methanol. After protein precipitation, the samples were centrifuged in a benchtop microcentrifuge at ~20,000 rcf for 10 minutes at 4°C. The supernatants were loaded onto Costar Spin-X 0.22 μm spin filters and centrifuged again at ~20,000 rcf for 1 minute at 4°C. The eluates were placed in a 96-well plate and injected onto an Agilent 1100 series high performance liquid chromatograph coupled to an Agilent G1956B single-quadrupole mass spectrometer. Chromatographic separation of the analytes was achieved using a HyperCarb 100 x 2.1 mm, 5 μm particle size column (Thermo Scientific). Mobile phase A was 100 mM ammonium acetate, pH 8.5, and mobile phase B was acetonitrile. The flow rate was 0.3 mL/min. The elution gradient started at 5% B, increased linearly to 30% B from 0.5 min to 4 min, held at 30% B until 6 min, then increased to 50% B by 8 min, held at 50% B until 9 min, then increased to 80% B by 9.5 min, held at 80% B until 10.5 min, then decreased to 5% B by 10.6 min, and the column was equilibrated at 5% B until 15 min post injection. The mass spectrometer was operated in electrospray ionization mode with positive polarity. Ions monitored were 664.1 (NAD+), 335.1 (NMN), and 425.9 (BMP) *m/z*. The fragmentor voltage was set to 120, 110, and 110 V for NAD+, NMN, and BMP, respectively. The capillary voltage was 3000 V.

For analyte calculations, the peak height ratios of NAD+/BMP, NMN/BMP, and NR/*d*_*3*_-NR were used, and the standard curves were fit to a second-order polynomial. The lower limit of quantitation (LLOQ) of NAD+, defined as the lowest standard with accuracy within ± 10% of actual value and precision with coefficient of variation < 10%, was 2.3 μM. The LLOQ of NMN was 1.5 μM, and the LLOQ of NR was 0.0156 to 0.0195 μM. Run-to-run quality control samples were considered to be acceptable when accurate to within ± 10% of expected concentration.

### Statistical analyses

A descriptive (non-compartmental) approach was used for the pharmacokinetic analysis of blood NR and NAD+ concentration-time data. The area under the blood concentration-time curve was calculated for each subject using the linear trapezoidal rule. The average concentration at steady state (C_ave,ss_) was calculated by dividing the AUC_0-12 hrs_ by the dosing interval of 12 hours.

Participant characteristics and baseline clinical laboratory data are presented as mean ± standard deviation (SD). Paired Student’s *t* tests assuming unequal variance were used to compare 1) baseline and Day 9 C_ave,ss_ values, 2) baseline and trough concentrations, and 3) baseline and peak concentrations for NR and NAD+. Similar statistical methods were used to compare baseline and Day 9 values for five secondary endpoints (serum levels of potassium, creatine kinase, glucose, uric acid and aspartate aminotransferase), as well as for ten additional variables of interest (systolic and diastolic blood pressures, body temperature, body weight, white blood cell count, hematocrit, hemoglobin, platelets, and serum levels of lactate dehydrogenase and alanine aminotransferase).

To calculate the correlation of changes in NAD+ concentrations with the NAD+ levels at baseline, the square of the Pearson correlation coefficient (R^2^) was calculated for (NAD+ C_ave,ss_ − NAD+ baseline concentration) vs. NAD+ baseline concentration. Similarly, the correlation of changes in concentrations of NR and NAD+ were calculated as the square of the Pearson correlation coefficient for (NR C_ave,ss_ − NR baseline concentration) vs. (NAD+ C_ave,ss_ − NAD+ baseline concentration).

All calculations were performed and graphs made using the statistical programming language R [13] and the R package ggplot2 [14].

## Results

### Participant characteristics

All eight enrolled participants completed the study. Participants were (mean ± SD) 33 ± 8 years of age and 75% female with the following self-reported ethnic distribution: 75% non-Hispanic white, 12.5% Asian and 12.5% African American.

### Safety assessments and other variables

No adverse events or side effects attributable to NR were reported during dose escalation of NR to 1000 mg orally twice daily.

Pre-specified laboratory safety endpoints were designed to evaluate possible laboratory abnormalities that might be seen with NR based on previous studies with NR (i.e., hyperkalemia) or that have been reported with either niacin and/or nicotinamide (i.e., myositis, hyperglycemia, hyperuricemia and hepatotoxicity). As shown in Table 1, no clinically significant changes were seen in any of the five pre-specified safety endpoints, i.e., potassium, creatine kinase, glucose, uric acid and alanine aminotransferase. Serum potassium decreased by an average of 0.4 mEq/L, which was statistically significant (*p* = 0.015) but the individual levels remain in normal range.

**Table 1:** Comparisons of pre-specified endpoints between baseline (Day 1) and Day 9. Parameter values reported as mean ± standard deviation.

N = 8 Participants	Baseline (Day 1)	Day 9	Mean difference	*p* value
Potassium (mEq/L)	4.1 ± 0.4	3.7 ± 0.1	–0.4	0.015
Glucose (mg/dL)	93 ± 7	97 ± 20	4	0.51
Uric acid (mg/dL)	5.0 ± 0.8	4.7 ± 0.6	–0.3	0.34
Creatine kinase (U/L)	120 ± 90	110 ± 60	–10	0.57
Alanine aminotransferase (U/L)	13 ± 4	14 ± 3	0.5	0.61

As shown in Table 2, a slight though statistically significant decrease was seen for hematocrit (mean difference = –2%, *p* = 0.005), hemoglobin (–0.4 g/dL, *p* = 0.04), and platelet count (–20,000/μL, *p* = 0.03). There were no significant changes in blood pressure, body temperature, body weight, white blood cell count, lactate dehydrogenase or aspartate aminotransferase. In addition, there were no significant changes in serum levels of sodium, chloride, urea nitrogen, creatinine, or in white blood cell differential (data not shown).

**Table 2:** Comparisons of other variables of interest between Day 1 and Day 9. Parameter values reported as mean ± standard deviation.

N = 8 Participants	Baseline (Day 1)	Day 9	Mean difference	*p* value
Systolic blood pressure (mm Hg)	120 ± 20	120 ± 20	0.5	0.88
Diastolic blood pressure (mm Hg)	75 ± 8	75 ± 7	0	1.00
Temperature (°C)	36.8 ± 0.3	36.8 ± 0.2	0	0.78
Actual Body Weight (kg)	70 ± 20	70 ± 20	–0.4	0.11
White blood cell count (thousands/μL)	4.3 ± 0.6	4 ± 1	0.2	0.62
Hematocrit (%)	40 ± 3	39 ± 3	–2	0.0054
Hemoglobin (g/dL)	13.4 ± 1.0	12.9 ± 1.1	–0.4	0.044
Platelet count (thousands/μL)	220 ± 40	200 ± 30	–20	0.031
Lactate dehydrogenase (U/L)	140 ± 20	140 ± 30	–2	0.78
Aspartate aminotransferase (U/L)	18 ± 4	18 ± 4	0.2	0.71

### Development of collection and processing methods for assay of NR and NAD+ in whole blood

During assay development before starting the study, NR was found to be highly unstable in blood, although the combination of 2.5 M citric acid and ACD solution improved NR stability in blood. At room temperature, the absolute peak heights of NR and the deuterated internal standard spiked into blood and processed as described in the methods section decreased by 8% to 14% over 30 min, but their ratios remained constant. While the latter observation means quantitation relying on signal ratio between analyte and internal standard is feasible in the event of *ex vivo* degradation or consumption, the decrease in absolute signal does limit our assay sensitivity (i.e., LLOQ). At the temperature of wet ice, spiked NR and deuterated internal standard appeared stable for 20 min, but decreased by 22% in 1 hour. This led to our current practice of processing the blood samples immediately to limit the lapse between blood draw and stabilization to < 5 min. NR in the sample becomes stable once blood proteins are precipitated, which allowed us to process the samples on the LC-MS/MS over the required run time. Also, storage data thus far indicate that NR blood samples are stable at –80°C for at least 3 weeks. With the resolution of NR’s instability problem, we were able to generate for the first time pharmacokinetic data on NR in human subjects.

It should also be noted that we did try to measure NR in the plasma fraction of a few blood samples obtained from volunteers following ingestion of NR, but failed to detect measurable levels of the riboside. We concluded that NR is concentrated in the cellular fraction of blood. Because NR’s instability in blood ex vivo, we decided to avoid the delay in separating cells from blood samples, and instead assay whole blood concentration.

Similar to NR, NAD+ is unstable in blood samples at room temperature. Measured levels of NAD+ spiked into thawed, room-temperature whole blood degraded by as much as 50% in 10 minutes. NAD+ degraded at a much slower rate (~3–4% decrease in 10 minutes) when blood samples were placed on wet ice. Also, NAD+ is stable in blood when stored at –80°C for at least 3 weeks. Hence, blood samples collected for NAD+ analysis were immediately placed on wet ice and then frozen in dry ice within 5 minutes of collection. Once blood proteins were precipitated with TCA, NAD+ in the supernatant remained stable for at least 48 hours while the samples were being processed on the LC-MS. Also, instability, at least over a few hours, was not observed when NAD+ was spiked into BSA rather than blood, suggesting that the disappearance of signal is not due to chemical instability.

### Pharmacokinetics of NR in whole blood

**Fig 3:**
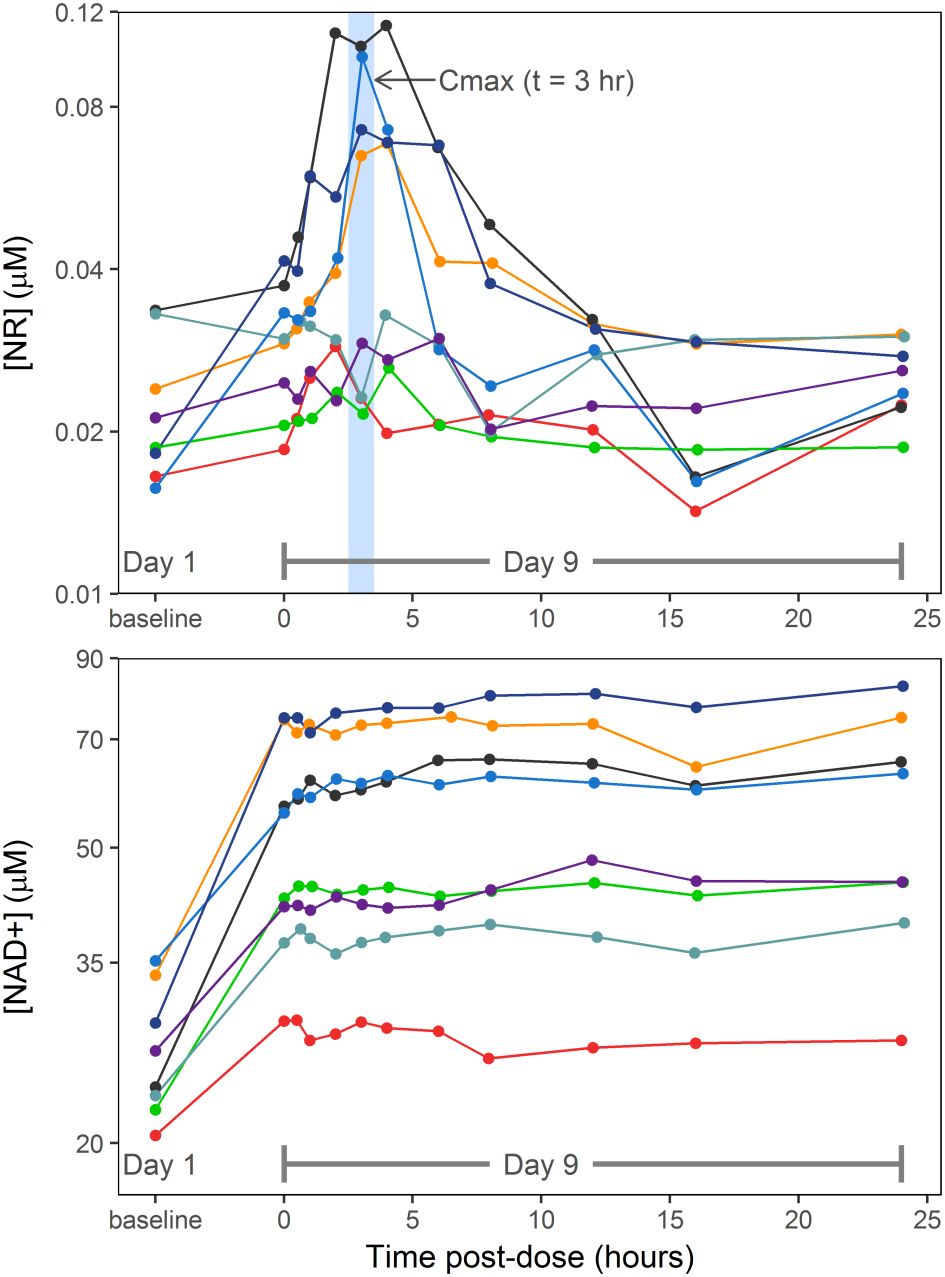
Concentration-time curves for NR (top) and NAD+ (bottom). Each subject is depicted in a different color with time points connected by a line. The y-axis depicts NR and NAD+ concentrations in μM, and the x-axis depicts values on Day 1 (baseline) and then time post-dose in hours on Day 9. The baseline time point was collected pre-dose on Day 1 of the trial.

Measurable levels of endogenous NR were observed in blood collected on Day 1 prior to treatment with NR supplement; basal levels of NR varied by 2.1-fold between the 8 subjects, from 0.0156 to 0.0336 μM (Fig 3).

All but one subject showed an increase in blood NR concentration from baseline to the Day 9 trough level with percent NR changes from baseline ranging from –10% to +127%. Following the Day 9 dose, the increase in blood NR concentration peaked at t = 3 hours (Fig 2) in half of the participants, while the other half had no obvious change in blood NR levels. The pharmacokinetics of blood NR appeared to have reached a steady state by the Day 9 dose (i.e., within two days of dosing at 1000 mg twice daily), as the trough NR level prior to the last dose was similar to the 12 hour post-dose NR level (0.031 ± 0.008 μM vs. 0.027 ± 0.004 μM, *p* = 0.1). For the four subjects with distinct C_max_, the mean blood NR concentration showed an exponential or log-linear decline from t = 3 hours to t = 12 hours, which afforded a regression estimate (± the standard error of the regression) for a first-order elimination rate constant of 0.26 ± 0.04 hr^-1^ and a corresponding half-life of 2.7 hours.

**Fig 4:**
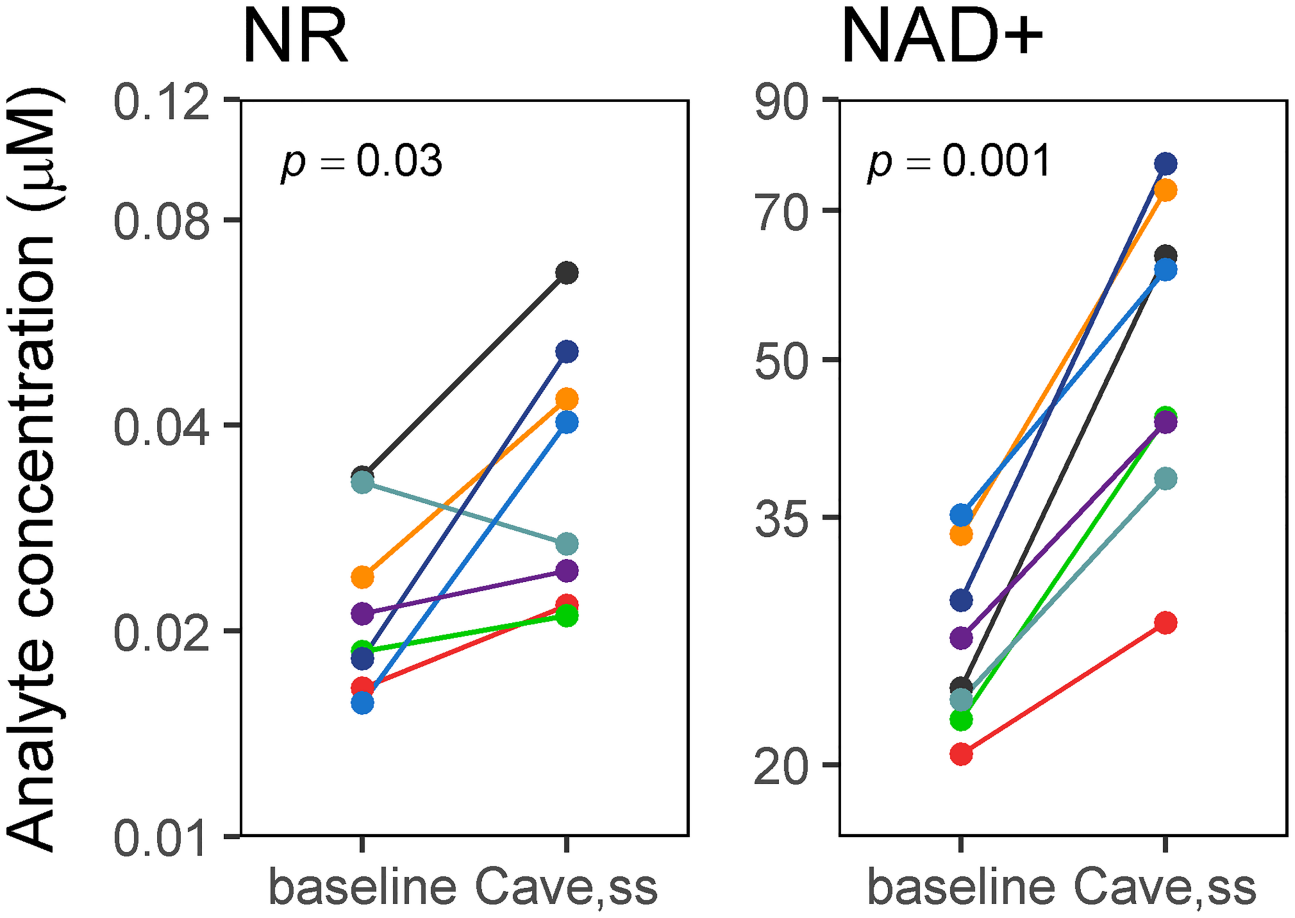
Line plots of concentrations (μM) of NAD+ and NR at Day 1 (baseline) and the average concentration at steady state (C_ave,ss_). Each subject is depicted in a different color and the time points are connected by a line. The *p* value for a paired Student’s *t* test is shown in the upper left corner of each plot.

To index the overall increase in blood NR concentration after the Day 9 dose compared to the baseline level, we calculated an average steady-state blood concentration based on the area under the blood concentration-time curve over the 12-hour dosing interval (i.e., C_ave,ss_). The C_ave,ss_ of NR was higher than baseline in seven of eight participants (mean difference = 0.014 μM, *p* = 0.03) (Fig 4).

Changes from baseline levels for NR are shown for C_ave,ss_ and two other pharmacokinetic variables (Baseline vs. Trough and Baseline vs. Peak) in Table 3. Peak concentrations were also significantly higher than the baseline concentration at the 95% confidence level (mean difference = 0.030 μM, *p* = 0.04).

**Table 3:** Comparisons of blood NR and NAD+ levels at baseline (Day 1) with trough, peak, C_ave,ss_ concentrations on Day 9. Values shown are mean ± standard deviation (n = 8); *p* values corrected for multiple testing using the Holm method.

	Baseline (Day 1)	Trough (time 0, Day 9)	Peak (time 3h, Day 9)	C_ave,ss_ (Day 9)	Trough vs. Baseline *p* value	Peak vs. Baseline *p* value	C_ave,ss_ vs. Baseline *p* value
NR (μM)	0.023 ± 0.007	0.029 ± 0.008	0.05 ± 0.03	0.04 ± 0.02	0.07	0.04	0.03
NAD+ (μM)	27 ± 5	50 ± 20	50 ± 20	50 ± 20	0.001	0.001	0.001

### Increase in steady-state blood NAD+ levels in response to NR

In the 8 participants, the mean circulating level of NAD+ at baseline (Day 1) was 27 ± 6 μM, consistent with values reported previously in healthy subjects [15, 16]. Nicotinamide adenosine dinucleotide (NADH) was not detected in blood. Nicotinamide mononucleotide (NMN), a known immediate precursor of NAD+, was detected in baseline (Day 1) blood samples of all subjects but was at or just above the assay lower limit of quantitation.

Notable elevations in blood NAD+ concentrations were seen on Day 9 compared to baseline on Day 1 in every subject (Fig 3). C_ave,ss_ for blood NAD+ was, on average, about 2-fold higher than baseline (range 1.34- to 2.66-fold), with a mean increase of 26.7 μM (*p* = 0.001). As a group, the rise in blood NAD+ (calculated as C_ave,ss_ − baseline) following NR treatment did not correlate with the baseline blood level on Day 1 (R^2^ = 0.27, *p* = 0.2). It should also be noted that blood NMN concentration did not show a measurable increase from baseline in any of the samples collected on Day 9 (data not shown). Trammell et al. [16] have also reported low levels of NMN in PBMCs of 12 healthy human subjects after single oral doses of NR at 100, 300 and 1000 mg; the mean maximum concentration at the 1000 mg dose was 2 μM (below the LLOQ of our assay) and was not significantly different from baseline.

**Fig 5:**
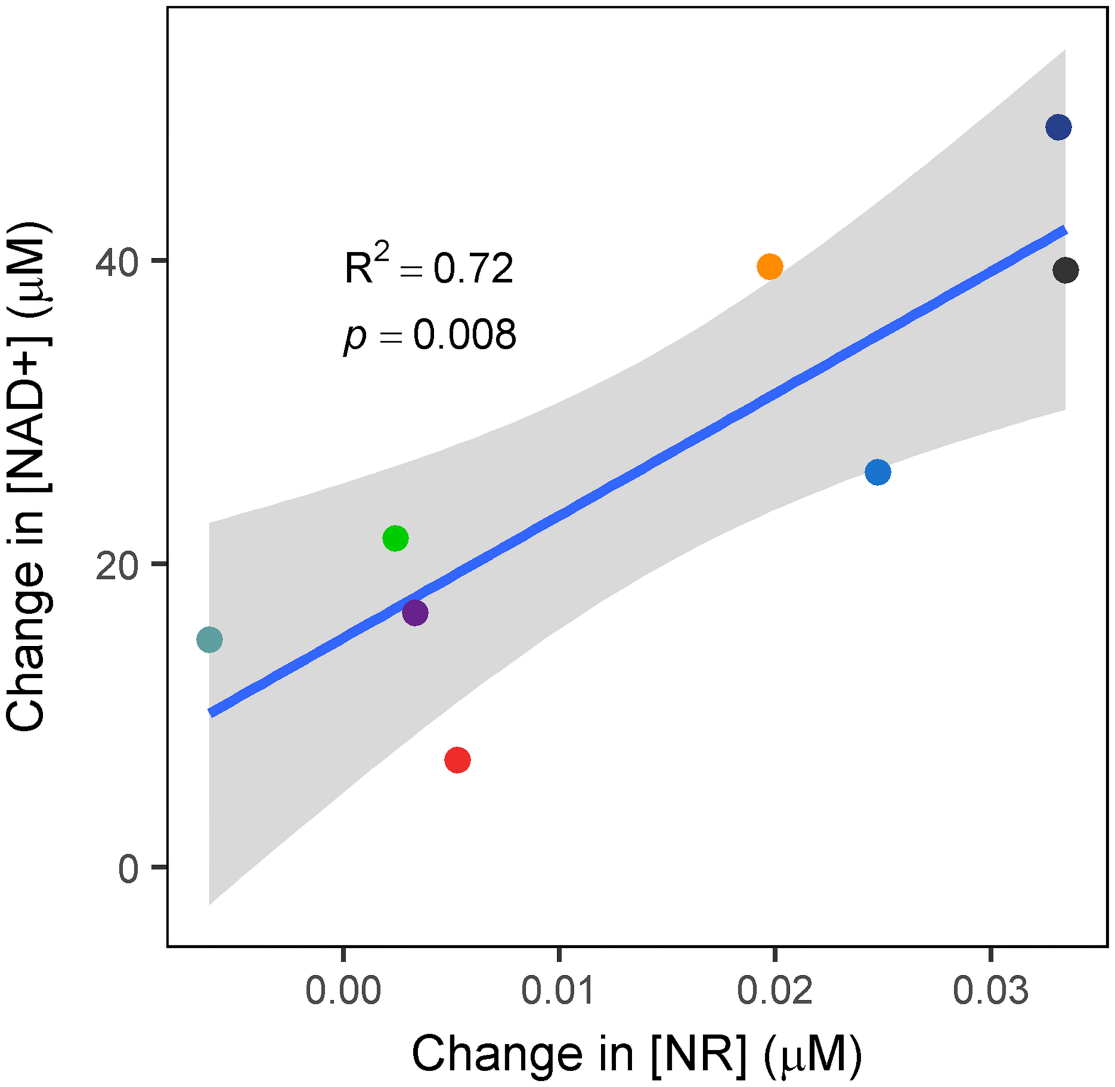
Correlation between absolute changes in NAD+ concentration versus NR concentration. Each subject is plotted as a different color. The blue line shows a linear regression of the change in NAD+ given the change in NR, and the shaded area shows the 95% confidence interval of that regression.

Changes in blood NR and NAD+ concentrations were correlated highly across the 8 subjects, with a Pearson correlation coefficient of 0.85 (R^2^ = 0.72, p = 0.008) (Fig 5).

Interestingly, while the Day 9 trough blood levels of NAD+ increased as compared to baseline in each of the eight participants, there was no observable further increase in blood NAD+ concentrations after the last dose of NR, i.e., measured NAD+ levels remained relatively constant during the 24-hour pharmacokinetic monitoring period.

## Discussion

This pilot study yielded several important findings. First, successful and reliable methods for collection, processing and measurement of NR and NAD+ in human blood were developed, overcoming the severe instability problems that have prevented previous investigations into the clinical pharmacokinetics of NR in blood or tissue. These methods allowed us to determine the pharmacokinetic profile of orally administered NR in healthy human volunteers.

Second, the study demonstrated that an NR dose of 1000 mg twice daily significantly increased steady-state, whole-blood levels of NAD+ in all study participants with individual increases ranging from 35–168% above baseline NAD+ levels.

Finally, the study demonstrated that measurable, biologic effects on NAD+ levels can be achieved in healthy volunteers at NR doses that are well-tolerated. Specifically, participants reported none of the serious side effects seen with similar doses of niacin, such as flushing, pruritus, hyperglycemia, hyperuricemia, or elevations in liver or muscle enzymes [17, 18]. Together, these findings support the feasibility of studying NR as a potential therapy for diseases in which mitochondrial dysfunction has been implicated.

At the time of our manuscript submission, Trammell et al. [16] reported a similar study on NAD+ metabolome in peripheral blood mononuclear cells (PBMC) in twelve healthy human subjects after single oral ingestion of NR at three different dose levels: 30, 100 and 1000 mg. Their findings are consistent with our experience and observations after 9 consecutive days of NR treatment at a final daily dose of 1000 mg. Over the 36 days of observation, Trammell et al. did not observe any serious adverse event and any event that was dose-related. Mean PBMC NAD+ concentration at 24-hours for pooled data across all three dose levels was significantly elevated compared to pre-dose concentration (p ≤ 0.03). Peak PBMC NAD+ concentrations rose by an average of 4 ± 2 μM at the 100 mg NR dose and reached a plateaued response average of 6.5 ± 3.5 μM at 300 and 1000 mg doses from a baseline average of 12 ± 3.3 μM. PBMC NAD+ concentrations for the 1000 mg dose reported by Trammell et al. are within range of the whole-blood NAD+ concentrations observed in this study. More importantly, the magnitude of increase in PBMC NAD+ concentration reported by Trammell et al. after a single 1000 mg dose of NR is quite comparable to our observed peak whole-blood concentration (see Table 2) after dose escalation to two final days of NR treatment at 1000 mg (+54% vs. 67%). It should be noted that Trammel et al. also profiled the NAD+ metabolomics in blood and urine collected from one male, 57-year old subject, who ingested 1000 mg of NR daily for 7 days. PBMC NAD+ concentration rose by 2.7-fold in this one subject, which matches the maximum increase in whole-blood NAD+ concentration we observed in our subjects. Trammell et al. did not include NR in their PBMC metabolome measurements.

There are a few additional findings with our NR and NAD+ data that warrant comment. The current study demonstrates that the apparent oral bioavailability of a 1000 mg dose of NR was highly variable among individuals. While half of the participants showed a significant increase (≥ 100%) in peak blood NR concentration during the pharmacokinetic portion of the study, the remaining subjects showed no or only modest (≤ +50%) changes in blood NR levels. The explanation for this inter-subject variability in post-dose NR levels is not obvious. The instability of NR in blood samples observed here as well as in prior studies [19] could be one contributing factor, although it cannot account for all the variability given the steps we have taken in minimizing instability during handling and processing of blood samples. Another possibility is that, based on its hydrophilicity (XLogP3 = –1.8) [20], NR is expected to exhibit low passive permeability across the human intestinal mucosa. Oral absorption of NR may rely on an active, mediated transport process that varies in active between individuals. It is also possible that NR is degraded to nicotinamide in the gut; nicotinamide is then absorbed and converted to NMN, which can further be converted to NAD+ or dephosphorylated to NR. If true, the degradation of NR to nicotinamide in the gut, which presumably involves purine nucleoside phosphorylase in mammalian and bacterial cells [21] may be a variable step involved in the oral intake of NR. Hence, investigation into the mechanism(s) of NR absorption and metabolism may afford insights into its variable oral bioavailability.

The highly significant correlation between changes in blood levels of NR and NAD+ supports the expectation that NR serves as a precursor to NAD+. Trammell et al. in their recent study [16] conducted comprehensive in vivo metabolomic investigations in one male human subject and in C57Bl6/J mouse, from which they were able to identify multiple pathways for conversion of NR to NAD+. One unexpected finding was a remarkable elevation (45-fold) in nicotinic acid adenine dinucleotide (NAAD), which was not thought to be en route for the conversion of NR to NAD+. Further labeling experiments in the mouse showed that at high NAD+ level deamidation of NAD+ to NAAD occurs in competition with NAD+ turnover to nicotamide. Trammell et al. further suggested that NAAD could serve as a metabolite reserve for NAD+ over time and a sensitive biomarker for increased NAD+ metabolism. We did not have an opportunity to measure NAAD in our subjects.

It is worth noting that the blood NAD+ response does not appear to correlate with the absorption pattern of NR; i.e., at steady state, blood NAD+ levels remain relatively constant over the 12-hour NR dosing interval, even though the blood NR concentration peaks around 3 hours and declines thereafter. This apparent dissociation in the temporal profile of blood NR and NAD+ may reflect much slower formation and/or turnover kinetics of NAD+ compared to the clearance kinetics of an exogenous load of NR. Alternately, the observation that 1000 mg NR given on Day 9 did not further increase NAD+ levels in any subject could imply that the maximal effect of NR on raising the NAD+ pool has been attained. Also, the rather short elimination half-life of NR observed in some of our subjects would suggest that frequent dosing throughout a day would be needed to prevent wide fluctuations in body levels of NR. However, given the sustained response of blood NAD+, twice-daily or even once-daily dosing of NR may be sufficient to achieve a desired clinical outcome.

While this study offers novel insights into NR as a nutritional supplement and as a potential therapeutic entity, it does have notable limitations. We enrolled healthy volunteers, who were predominantly white and female. It remains to be determined whether there are gender, age and/or ethnic group-specific differences in NR and NAD+ metabolism; thus the results of this study might not apply generally to diverse patient populations. Similarly, different disease states and pre-existing metabolic derangements also could affect the pharmacokinetics and pharmacodynamics of NR. While the present study indicates the feasibility of elevating the circulating pool of NAD+ by NR treatment; however, NR’s effects on intracellular NAD+ levels and the NADH/NAD+ ratio at critical tissue sites remain unknown. These issues, which are critical in determining the therapeutic potential of oral NR supplementation, must wait for further clinical studies in specific patient populations.

In summary, this study both reports the successful development of protocols for assaying NR and NAD+ levels in whole blood and demonstrates that orally administered NR increases the circulating pool of NAD+ in healthy, human volunteers without apparent clinical side effects or significant changes in relevant laboratory variables. These findings demonstrate the feasibility of translating NR supplementation to a therapeutic reality, as animal studies have shown that NAD+ precursors are beneficial in combatting inflammation, ischemic/hypoxic injury, oxidative stress, and cancer [11, 12, 22, 23]. In particular, NR appears to be well positioned as a novel therapy for systolic heart failure, where impaired mitochondrial oxidative phosphorylation and disturbed NAD+ metabolism are contributory factors for its development and progression [7–9, 12]

## Supporting information

S1 File TREND checklist 10 12 2016. (PDF)

S2 File NR PK study FINAL protocol 7-21-15. (PDF)

S3 File NR PK lab protocol 10_09 FINAL. (PDF)
