# Supplementary material for: An open-label, non-randomized study of the pharmacokinetics of the nutritional supplement nicotinamide riboside (NR) and its effects on blood NAD+ levels in healthy volunteers
Source: PLoS One. 2017 Dec 6;12(12):e0186459. doi: 10.1371/journal.pone.0186459 (PMC5718430; doi:10.1371/journal.pone.0186459)
Supplement: S2 File — (PDF) [file pone.0186459.s002.pdf]

Nicotinamide Riboside PK Study: CRC Protocol
FINAL 7/21/2015

1. Protection of Human Subjects

1.1. Risks to the Subjects

(a) Human Subjects Involvement and Characteristics: This proposed pharmacokinetic Study in healthy volunteers will be conducted at the University of Washington, with laboratory studies to be performed at the Analytical Core of the UW Nutrition and Obesity Center (NORC) and the UW Department of Pharmaceutics (NR assays). The IRB application for this Study will be obtained using the Just-In-Time approach. The inclusion and exclusion criteria of this Study are as followings:

Inclusion Criteria:

- Healthy volunteers of both genders, ages 21-50, recruited from the community.
- Ability to undergo Study procedures, including scheduled visits, blood draws and overnight stay on the UW GCRC
- Willingness/ability to provide informed consent

Exclusion Criteria:

- Current smoking
- Pregnant (or likely to become pregnant) women
- Receiving concurrent medications or supplements
- Any history of liver, renal, cardiovascular, endocrine or neurological disease
- Known allergies to niacin or nicotinamide
- Unwillingness to refrain from drinking alcohol during the duration of the Study
- Inability to perform Study visits or procedures (e.g., overnight stay on the UW GCRC)
- Unwillingness/inability to provide informed consent

(b) Study Procedures and Randomized Therapy:

This Study is a single-center, prospective, Study of the safety and pharmacokinetics of NR in 8 healthy volunteers.

Screening: Prospective, eligible participants will undergo a screening visit to obtain demographic information, medical history, physical examination, and current medical treatments (Day 1). If eligibility is confirmed, they will be entered into the Study

Clinical and follow-up visits:

<u>Screening visit (Day 1)</u>

- Demographic information, medical history, physical examination, current medical treatments
- Obtain a baseline 12 lead resting ECG
- Fasting blood draw: Two 5mL EDTA tube, please process one of these samples as a buffy coat (CBC with WBC differential and platelets, NADH/NAD levels, NR level-buffy coat) and 10mL “red top” tube [serum chemistry panel (sodium, potassium, chloride, glucose, blood urea nitrogen and creatinine), uric acid, CPK, AST and ALT, and LDH and serum NR level]
- Eligible participants will begin NR uptitration protocol, with NR doses of:
    - 125 mg twice daily on Days 1-2
    - 250 mg twice daily on Days 3-4
    - 500 mg twice daily on Days 5-6
    - 1000 mg twice daily on Days 7-8
- Collect urine samples in two 10ml conical vials: if subject is a female, run urine pregnancy test and freeze the remaining urine sample at -70 degrees

<u>Safety Labs visit (Day 2)</u>

- 10mL “red top” tube [serum chemistry panel (sodium, potassium, chloride, glucose, blood urea nitrogen and creatinine)]

<u>Overnight UW GCRC visit (Day 9)</u>

- Admission to the UW GCRC for overnight stay
- Demographic information, medical history, physical examination, current medical treatments
- Obtain a 12 lead resting EKG
- Fasting blood draw: Two 5mL EDTA tube, please process one of these samples as a buffy coat (CBC with WBC differential and platelets, NADH/NAD levels, NR level-buffy coat) and 10mL "red top" tube [serum chemistry panel (sodium, potassium, chloride, glucose, blood urea nitrogen and creatinine), uric acid, CPK, AST and ALT, and LDH and serum NR level]
- Record of adverse events, if any
- Patients then will begin pharmacokinetic Study:
    - Fasting blood draw: 10mL EDTA tube (CBC with WBC differential and platelets, NADH/NAD levels) and 10mL "red top" tube [serum chemistry panel (sodium, potassium, chloride, glucose, blood urea nitrogen and creatinine), uric acid, CPK, AST and ALT, and LDH and serum NR level]
    - Subjects then will receive the last 1000 mg dose of NR at t=0.
    - Blood samples will be collected at t= 0.30, 1, 2, 3, 4, 6, 8, 12, 16 and 24 h (8mL "red top" tube at each timepoint).
    - Serum samples will be frozen at -70°C until assayed for NR using LCMS/MS.

Every attempt should be made to complete the visits during the defined window periods. A final follow-up visit is required for all participants.

Laboratory results:

Participants will be blinded to the results of laboratory studies obtained as a part of this Study.

(c) Sources of Materials: Data will be collected throughout the Study from participants on their health status and concomitant medications. Clinical, demographic and laboratory values collected as a part of the Study will be stored in a multidimensional database at the UW Clinical Atherosclerosis Research Laboratory (CARL). All lab samples are identified by a Study number only. Individually identifiable data is recorded in individual Study charts for each participant, and in a password-protected Study database. Individually identifiable data will be accessible only to the Study investigators.

(d) Potential Risks: Participants may experience discomfort and bruising from blood draws. Rarely, an infection could develop.

This is a Study of a nutritional supplement, NR. A warm flushing sensation may occur when first taking this supplement. We will monitor for skin rash, increased blood sugar, or cause mildly abnormal hepatic function. "Safety labs" will monitor for electrolyte abnormalities (especially hyperkalemia) and changes in glucose, uric acid and CBC, as well as renal or hepatic dysfunction.

1.2. Adequate Protection Against Risk

Recruitment and Informed Consent. Recruitment for this proposed Study will be conducted according to the IRB policies at the University of Washington, as well as HIPPA policies. Potential Study participants will be identified at the Screening visit. Screening of potential participants will be conducted on-site to bypass security concerns about the electronic transfer of participant information. A simple question - "Would you like to participate in a Study that looks at whether a nutritional supplement is safe and well-tolerated in participants with heart failure?" will be presented to the participants. Only if the participant expresses any interest will we continue Study recruitment.

To the participants who are interested in the Study, investigators will explain the Study's purpose and design and what would be required of participants. A series of questions will be asked to make sure the participant meet eligibility requirements. Then, the informed consent will be conducted by an investigator or a Study coordinator. Participants will be encouraged to ask questions and to share the information with their regular doctor. A copy of this consent will be given to the participants; the original will be placed in the participant's Study chart.

Protection Against Risks. All access to individually identified participant data is limited to the investigators who are directly involved in this research Study. Study charts and password-protected databases are confidential and are not released to outside parties except at the participant's request. Identifiable information is not released to any other department or person in the University of Washington. All blood samples for laboratory analysis are ONLY identified by a Study number. The Study charts containing paper forms will be

stored in the locked file cabinets in the research clinics or offices where they only can be accessed by the investigators. Each participant will receive a unique Study number. The Study number is linked to the participant's personally-Identifiable information in the Study database ONLY. The Study number, without personally-identifiable information, will be used to code blood samples for laboratory analysis and to perform data entry and linking. Furthermore, personally-identifiable information is not released to any other department or person in the University of Washington.

Notification of Participants and Their Physicians. Study participants and their primary care or physicians will be notified if any laboratory abnormalities develop. We will leave the further clinical follow-up and treatment plans to be made by the participants' physicians. Participants will not be notified of the results of other laboratory studies performed specifically for this Study.

1.3. Potential Benefits of the Proposed Research to the Participants and Others

There is no anticipated benefit for individual participants in this Study.

1.4. Importance of the Knowledge to be Gained

If the Study can demonstrate that nicotinamide riboside is safe and well-tolerated in healthy volunteers, this will form the basis of a double-blind, placebo-controlled follow-on Study of NR in participants with systolic heart failure Thus, the Study may provide information necessary to further explore the clinical utility of this nutritional supplement in the treatment of systolic heart failure, a large and growing population of high risk participants for whom treatment, at present, is less than adequate.

2. Data and Safety Monitoring Plan

A Data Safety Monitoring Board (DSMB) will be instituted for this Study in order to ensure its ongoing safety and to oversee the Interim Analysis after 4 participants have completed the Study. Recommendation for trial continuation will be guided by monitoring boundaries at interim analyses of safety evaluations all safety data reviews. Safety review meetings will be held by the DSMB prior to Study initiation, at which time the DSMB will develop any formal "stopping rule(s)" for the Study. The DSMB will meet two additional times: after completion of 4 and 8 30 Study participants. Safety data will include pre-specified evaluation of parameters for blood glucose, myopathy, hepatotoxicity, renal function, or other possible clinical side effects such as gout, as requested by the DSMB. Enrollment to the Study will continue throughout the scheduled meetings of the DSMB. The three-member, Study DSMB Roster is provided below.

1. **Jeffrey L. Probstfield, MD.** (Chair)
   Director, Clinical Trials Service Unit and Professor of Medicine (Cardiology)
   University of Washington School of Medicine
2. **W. Robb MacLellan, MD**
   Professor of Medicine and Head, Division of Cardiology
   University of Washington
3. **Kelley Branch, MD**
   Deputy Director, Clinical Trials Service Unit and Assistant Professor of Medicine (Cardiology)
   University of Washington School of Medicine
