# Supplementary material for: An open-label, non-randomized study of the pharmacokinetics of the nutritional supplement nicotinamide riboside (NR) and its effects on blood NAD+ levels in healthy volunteers
Source: PLoS One. 2017 Dec 6;12(12):e0186459. doi: 10.1371/journal.pone.0186459 (PMC5718430; doi:10.1371/journal.pone.0186459)
Supplement: S3 File — (PDF) [file pone.0186459.s003.pdf]

NR Pharmacokinetics
Clinical Research Center
MANUAL of PROCEDURES
For Specimen Collection and Processing

For Blood Collection:

- Collection tubes
  - 5 mL Purple-top
  - 5 ml Blue-top
  - 3.6 ml White cap study tube (NR)
  - 4.0 ml Orange cap (NADH/NAD+)
  - 10 ml Red-top

For Specimen Identification:

- Patient study ID, date and time of collection, and initials of lab personal collecting specimen

Other Equipment and Supplies

- Specimen Biohazard bag
- Wet Ice
- Dry Ice
- -80 degree freezer

SPECIMEN COLLECTION FOR NR PK Study

NOTE: Due to the sensitivity of these samples the instructions below must be followed to assure that the quality of these samples be maintained. Any deviation from the instructions below may have extreme compromising effects on the samples thus compromising the accuracy of the analysis being performed. If you have any questions regarding these procedures it is imperative that you call the Study Investigator, Sophia Airhart at 541-968-8199.

Schedule of Assessments

VISIT: Baseline (Day 1)

1. **Obtain demographic information, medical history, physical examination, current medical treatments**
2. **Obtain a baseline 12 lead resting ECG**
3. **Obtain Labs**

Analyses	Fasting Condition	Blood Collection Tube	Visual Reference	Transfer Plasma/Serum to Labeled Tubes and immediately put on ice
CBC with differential	Fasting	5ml **Purple**-top (Lavender Hemogard)	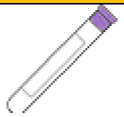	1.Collect 3 ml of blood and **MIX WELL**<br>2.Put on wet ice
Mixing tube	Fasting	5ml **Blue** top (sodium citrate tube)	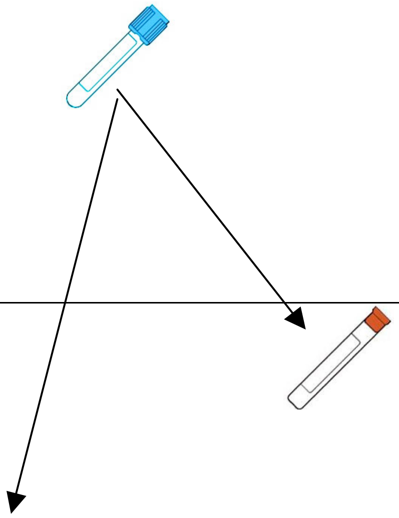	1.Collect 4ml whole blood<br>2.After inversion mixing the blue tube, pipet exactly 0.5ml into 4 white cap study tubes<br>3.Transfer the remaining 2ml of blood into the orange capped cryo tube
NADH/NAD+	Fasting	4ml **Orange** top	Pipet 0.5ml each into 4 white top tubes	1.Collect 4 ml of blood and **MIX WELL**<br>**2.Transfer 2ml of blood into separate NR tube**<br>**3.Immediately**, **freeze on dry ice** and **transfer to -80 degree freezer**
NR Level	Fasting	3.6ml Study Tube (white cap) with prefilled preservative	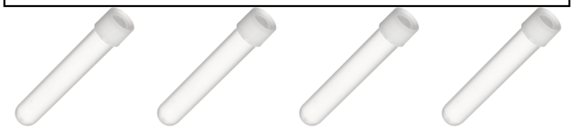	1.After mixing the blue tube, Pipette 0.5ml into 4 white cap tubes from blue top tube and **VORTEX WELL**<br>2.**Immediately**, **freeze on dry ice** and **transfer to -80 degree freezer**
Serum chemistry panel, uric acid, CPK, AST and ALT, and LDH	Fasting	10ml **Red** top	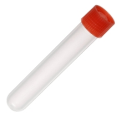	Fill tube and immediately put on wet ice

2. **Collect urine samples in two 10ml conical vials: if subject is a female, run urine pregnancy test and freeze the remaining urine sample at -70 degrees**

10 ml conical vial, self standing, sterile

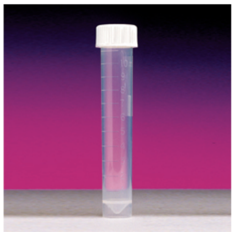

How do I collect specimens?

It is presumed that all personnel performing this work have been trained in proper blood collection procedures, listed below are just some important reminders.

As you draw blood, remember to:

- Mix each purple-top and blue top blood tube **8-10 times immediately after collection** by inverting the tube gently and evenly.
- **Avoid under-filling** the vacutainer tubes.

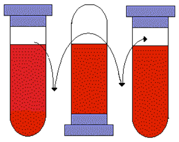

= 1 inversion

VISIT 2: Safety Labs Visit (Day 2)

Analyses	Fasting Condition	Blood Collection Tube	Visual Reference	Transfer Plasma/Serum to Labeled Tubes and immediately put on ice
Serum chemistry panel	Not Fasting	10ml Red top	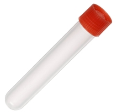	Fill tube and immediately put on ice

VISIT 3: Overnight UW GCRC Visit (Day 9)

1. **Obtain demographic information, medical history, physical examination, current medical treatments**
2. **Obtain a 12 lead resting ECG**
3. ***<u>Subjects will receive the last 1000 mg dose of NR at t=0</u>***
   - Subjects then will begin pharmacokinetics study
   - Collect the 4 tubes as instructed below at time =0
   - At 30 minutes, 1 hr, 2 hr, 3 hr, 4 hr, 6 hr, 8 hr, 12 hr, 16hr, and 24hr a 4ml into the BLUE tube should be collected and distributed into the orange and white top tube as previously described

**Analyses**	**Fasting Condition**	**Blood Collection Tube**	**Visual Reference**	**Transfer Plasma/Serum to Labeled Tubes and immediately put on ice**
CBC with differential<br><br>**Collect at t=0**	Fasting	5ml **Purple**-top (Lavender Hemogard)	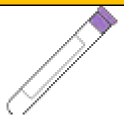	1.Collect 3 ml of blood and **<u>MIX WELL</u>**<br>2.Put on wet ice
Mixing tube<br><br>Collect at:<br>**t=0**<br>**t=30min**<br>**t=1 hr**<br>**t=2 hr**<br>**t=3 hr**<br>**t=4 hr**<br>**t=6 hr**<br>**t=8 hr**<br>**t=12 hr**<br>**t=16 hr**<br>**t=24 hr**	Fasting	5ml **Blue** top (sodium citrate tube)	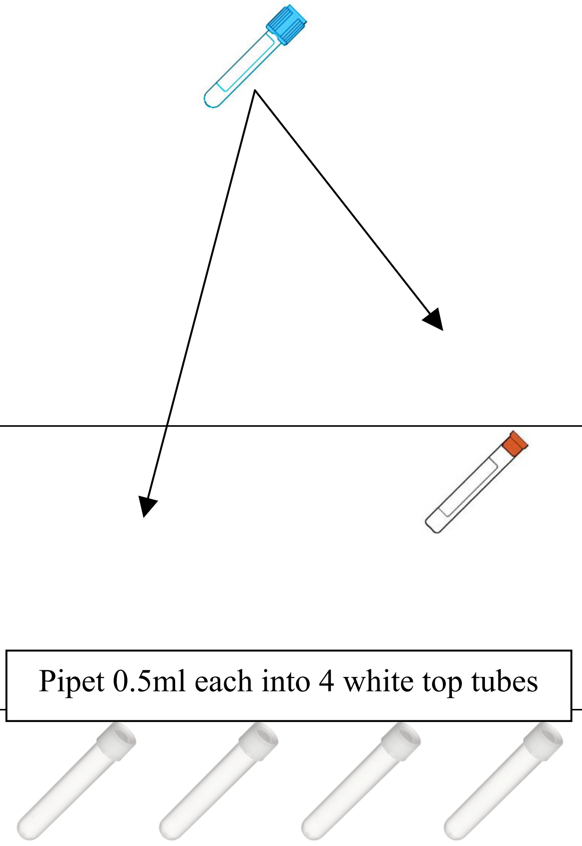	1.Collect 4ml whole blood<br>2.After inversion mixing the blue tube, pipet exactly 0.5ml into 4 white cap study tubes<br>3.Transfer the remaining 2ml of blood into the orange capped cryo tube
NADH/NAD+	Fasting	4ml **Orange** top		1.Collect 4 ml of blood and **<u>MIX WELL</u>**<br>**2.Transfer 2ml of blood into separate NR tube**<br>3.**Immediately**, **<u>freeze on dry ice</u>** and **<u>transfer to -80 degree freezer</u>**
NR Level	Fasting	3.6ml Study Tube (white cap) with prefilled preservative		1.After mixing the blue tube, Pipette 0.5ml into 4 white cap tubes from blue top tube and **<u>VORTEX WELL</u>**<br>2.**Immediately**, **<u>freeze on dry ice</u>** and **<u>transfer to -80 degree freezer</u>**
Serum chemistry panel, uric acid, CPK, AST and ALT, and LDH<br><br>**Collect at t=0**	Fasting	10ml **Red** top	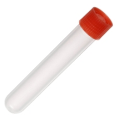	Fill tube and immediately put on wet ice
